# Microfluidic Reactors for Plasmonic Photocatalysis Using Gold Nanoparticles

**DOI:** 10.3390/mi10120869

**Published:** 2019-12-11

**Authors:** Huaping Jia, Yat Lam Wong, Aoqun Jian, Chi Chung Tsoi, Meiling Wang, Wanghao Li, Wendong Zhang, Shengbo Sang, Xuming Zhang

**Affiliations:** 1 MircoNano System Research Center, College of Information and Computer Science, Taiyuan University of Technology, Taiyuan 030000, China; jiahuaping312@gmail.com (H.J.); mlwang_001@163.com (M.W.); 15700747305@163.com (W.L.); wdzhang@tyut.edu.cn (W.Z.); sunboa-sang@tyut.edu.cn (S.S.); 2 Department of Applied Physics, The Hong Kong Polytechnic University, Hong Kong 999077, China; 17900203r@connect.polyu.hk (Y.L.W.); terry.cc.tsoi@connect.polyu.hk (C.C.T.)

**Keywords:** microfluidics, microreactors, gold nanoparticles, localized surface plasmon resonance, visible light photocatalysis

## Abstract

This work reports a microfluidic reactor that utilizes gold nanoparticles (AuNPs) for the highly efficient photocatalytic degradation of organic pollutants under visible light. The bottom of microchamber has a TiO_2_ film covering a layer of AuNPs (namely, TiO_2_/AuNP film) deposited on the F-doped SnO_2_ (FTO) substrate. The rough surface of FTO helps to increase the surface area and the AuNPs enables the strong absorption of visible light to excite electron/hole pairs, which are then transferred to the TiO_2_ film for photodegradation. The TiO_2_ film also isolates the AuNPs from the solution to avoid detachment and photocorrosion. Experiments show that the TiO_2_/AuNP film has a strong absorption over 400–800 nm and enhances the reaction rate constant by 13 times with respect to the bare TiO_2_ film for the photodegradation of methylene blue. In addition, the TiO_2_/AuNP microreactor exhibits a negligible reduction of photoactivity after five cycles of repeated tests, which verifies the protective function of the TiO_2_ layer. This plasmonic photocatalytic microreactor draws the strengths of microfluidics and plasmonics, and may find potential applications in continuous photocatalytic water treatment and photosynthesis. The fabrication of the microreactor uses manual operation and requires no photolithography, making it simple, easy, and of low cost for real laboratory and field tests.

## 1. Introduction

Water pollution is destroying the environment and has become a global challenge, forcing an urgent call for safe and effective methods to degrade and remove harmful organics from polluted water [1]. Photocatalysis typically utilizes semiconductor materials to absorb light and excite electron-hole pairs for further chemical reactions [2], offering a promising solution for solar energy conversion and environmental remediation [3]. As one of the prominent semiconductor photocatalysts, titanium dioxide (TiO_2_) has drawn considerable attention in the mineralization of harmful organic substances thanks to its superior properties of nontoxicity, high chemical stability, high photostability, abundance in nature, and low cost [4,5,6]. However, the photocatalytic efficiency of TiO_2_ in visible light is low as it is limited by its wide bandgap (3.2 eV).

Over the past two decades, noble metal nanoparticles (NPs) have been used to improve the efficiency of wide-bandgap photocatalytic materials such as TiO_2_ and ZnO [7,8]. The NPs of noble metals (e.g., Au, Ag, Pd, and Pt) exhibit a fascinating optical property of localized surface plasmon resonance (LSPR) due to the collective oscillation of free electrons in response to the excitation of irradiant light. The LSPR effect can drastically enhance the visible response of TiO_2_ photocatalysis for solar energy capture, environmental redemption, and selective organic photosynthesis [5,7,9,10]. Moreover, the direct physical contact of the noble metal NPs and the TiO_2_ photocatalysts would form a Schottky junction to suppress the recombination of electron-hole pairs [8,11].

Typical photodegradation systems involve the suspension of TiO_2_ nanopowders in an aqueous solution of a bulky container. With the stirring, the TiO_2_ nanopowders have full contact with the dissolved organic pollutants, resulting in a large specific surface area (SSA, defined as the total surface area per unit of mass) and high photodegradation efficiency. However, the suspended TiO_2_ nanopowders absorb and scatter light, causing rapid decay, and thus an uneven distribution of the irradiant light. What is more problematic is the requirement of post processing, namely the nanopowders have to be separated from the solution after the reaction [12,13,14]. To avoid these problems, immobilized systems have been developed to fix the TiO_2_ photocatalysts on a support, but they tend to have a small SSA and low efficiency [15].

Microfluidic reactors have attracted much attention and have been proposed to tackle the drawbacks of photocatalytic processes [14,16,17,18,19,20]. They inherit many advantages from microfluidics technology, such as small dimensions, high surface-to-volume (S/V) ratio, easy control of flow rates, short molecular diffusion distance, rapid reaction speed, high reaction efficiency, low reagent consumption, fast heat dissipation, uniform illumination of light, as well as potential portability and disposability [2,16,21,22,23]. Although a single microreactor has very limited output (~1–100 L/h), the throughput can be scaled up by connecting many devices in parallel, sizing up the reactor dimensions, and even stacking multiple layers of the same devices [14,24,25,26].

**Figure 1:**
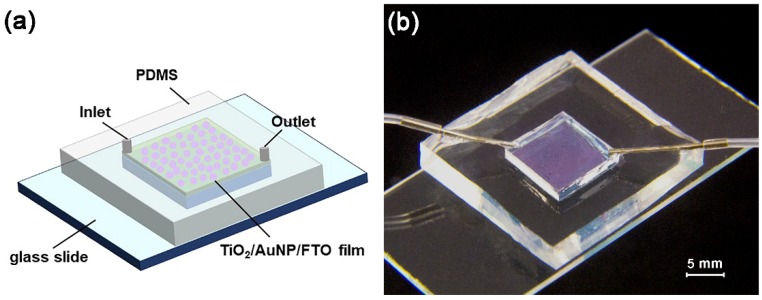
3D diagram and photo of the TiO_2_/AuNP microreactor.

**Figure 2:**
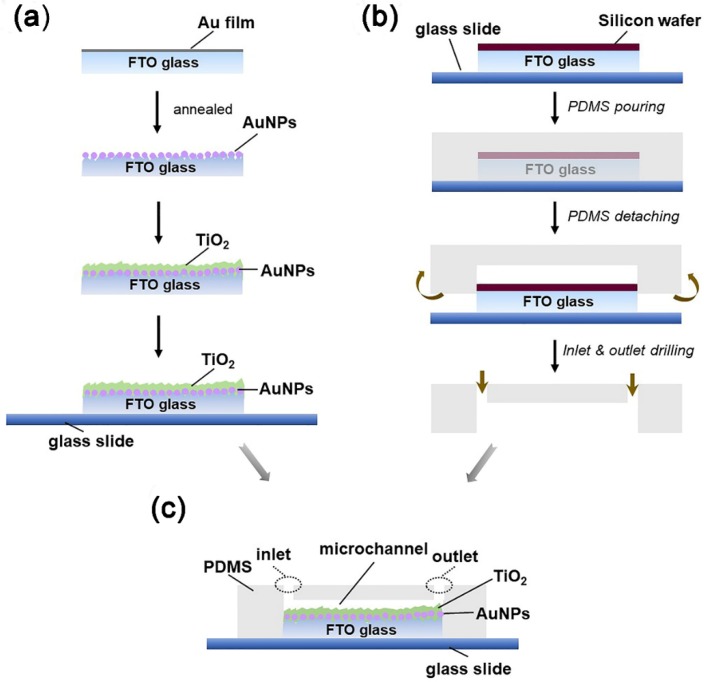
Fabrication and integration of the microreactors. (**a**) Fabrication process of the TiO_2_/AuNP film; (**b**) non-photolithographic manual molding of the polydimethylsiloxane (PDMS) cover; and (**c**) cross-sectional view of the microreactor after the PDMS cover is bonded on the TiO_2_/AuNP film.

Based on the above considerations, this work will incorporate the plasmonic effect into the microreactors so as to exert the full power of both for the enhancement of photodegradation efficiency. The microreactor is bonded on an FTO glass substrate to have a planar reaction chamber, the bottom part of which is decorated by gold nanoparticles (AuNPs) and then covered by a thin TiO_2_ layer (Figure 1 and Figure 2). Hereafter, this functional film is called the TiO_2_/AuNP film. The AuNPs have strong, tunable surface plasmon resonance properties in the visible light region and show strong scattering and absorption enhancement [5]. The TiO_2_ film serves dual purposes. The first of these is that it has direct contact with the solution in the microchamber for photodegradation. Further, it isolates the AuNPs from the solution, avoiding the common problems of detachment, photocorrosion, and thus low stability of AuNPs that are often found in previous work [27,28]. The use of FTO glass rather than more commonly used silica glass as the substrate is because the FTO glass has a rough surface, which helps to increase the SSA of the TiO_2_/AuNP film. Detailed experimental studies will be carried out to quantify the photodegradation efficiency.

## 2. Materials and Methods 

### 2.1. Fabrication of Au Nanoparticles and TiO_2_ Films

The procedures are illustrated in Figure 2a. Prior to fabrication, FTO glass (thickness 2.2 mm) is cleaned by acetone, ethanol, and water, successively, in an ultrasonic bath for 5 min. The dried substrate is transferred to an e-beam system (JSD500 Electron Beam, JS Vacuum, Auhui, China) to deposit a 3-nm thick Au layer onto the conductive side of the FTO glass. Then, the sample is annealed in 480 °C for 1.5 h to form the AuNPs on the FTO surface. Subsequently, a 15-nm thick TiO_2_ layer is deposited by atomic layer deposition (ALD, Cambridge NanoTech) with the TiCl_4_ and H_2_O used as precursors in the N_2_ atmosphere. The deposition rate of TiO_2_ is estimated to be 0.55 Å per cycle in 100 °C and the thickness of TiO_2_ film is controlled by the deposition cycles (number = 273). For comparison, a bare TiO_2_ film is also fabricated by the ALD. The prepared TiO_2_ films are annealed in 500 °C for 1 h to crystallize into the anatase phase. In this work, the TiO_2_ films have the same thickness of 15 nm if not specified.

### 2.2. Fabrication of Microreactors

The fabrication of microreactors does not involve photolithography, instead it is all done by manual operation, as shown in Figure 2b. Here, the microreactor consists of two functional parts: the TiO_2_/AuNP film and the PDMS cover. The former is fabricated on the FTO glass as stated above, which is then cut into small dies (dimensions 10 × 10 × 2.2 mm) and adhered to a glass substrate by applying a thin layer of UV-curable adhesive (NOA81, MicroChem, Westborough, MA, USA) with a UV exposure for 1 min. The latter is fabricated by replicating a mold using PDMS. The mold is prepared according to the following three steps:

1. A piece of FTO block (dimensions 10 × 10 × 2.2 mm) is cut from an FTO glass.
2. Another piece of thin silicon wafer (thickness = 0.46 mm) of the same footprint is mounted on top of the FTO block by NOA81.
3. The silicon/FTO block is further adhered to a glass slide by NOA81.

In this way, the mold is a silicon/FTO block with the dimensions of 10 × 10 × 2.7 mm. In the PDMS replication process, a PDMS polymer base and curing agent (Sylgard 184, Dow Corning Corporation, Midland, MI, USA) are mixed at a ratio of 10:1 by weight before being cast onto the silicon/FTO mold and baked at 80 °C for 1 h. Next, the PDMS layer is cut and peeled off, obtaining a reverse pattern of the mold on one side of the PDMS slab (dimensions 20 × 20 × 4.5 mm). The reverse pattern is a pothole with the dimensions 10 × 10 × 2.7 mm. Inlet and outlet holes are punched in the PDMS slab as well. Finally, the TiO_2_/AuNP film and the PDMS slab are bonded together using NOA81 by carefully aligning the reaction microchamber to the TiO_2_/AuNP film (Figure 2c). After the attachment of soft tubes to the inlet and outlet holes, the microreactor is read for experimental tests. As stated above, the TiO_2_/AuNP film together with the FTO substrate has a thickness of 2.2 mm and the pothole has a depth of 2.7 mm, therefore the reaction chamber has a height of 0.5 mm.

### 2.3. Photocatalytic Degradation Experiment

The photocatalytic activity of TiO_2_/AuNP microreactor is investigated under a simulated solar source (AM 1.5G, 300 mW/cm^2^) equipped with a UV-cutoff filter to obtain visible light (λ > 420 nm). In all experiments, the prepared sample is placed at a distance 15 cm away from the light source. Methylene blue (MB) is used as a model chemical to quantify the photodegradation performance [15]. The MB solution (concentration 5 × 10^−5^ M) is introduced through the inlet of the microreactor by a syringe pump (Longer). The degraded MB solution is collected from the outlet of the device. The degradation of MB can be evaluated by monitoring the change of MB’s absorbance at the wavelength of 664 nm using a UV-vis spectrophotometer (Perkin-Elmer Lambda 950). The absorption spectra of the fabricated films are investigated using the same UV–vis spectrophotometer, but with an integrated sphere. The atomic force microscopy (AFM) images of nanoparticles are collected in air in a tapping mode by using a silicon cantilever (SI-DF20, Seiko Instruments, Japan). The scanning electron microscopy (SEM) images are obtained using JEOL JSM-6335F (JEOL, Japan).

## 3. Results and Discussion

### 3.1. Material Characterization

**Figure 3:**
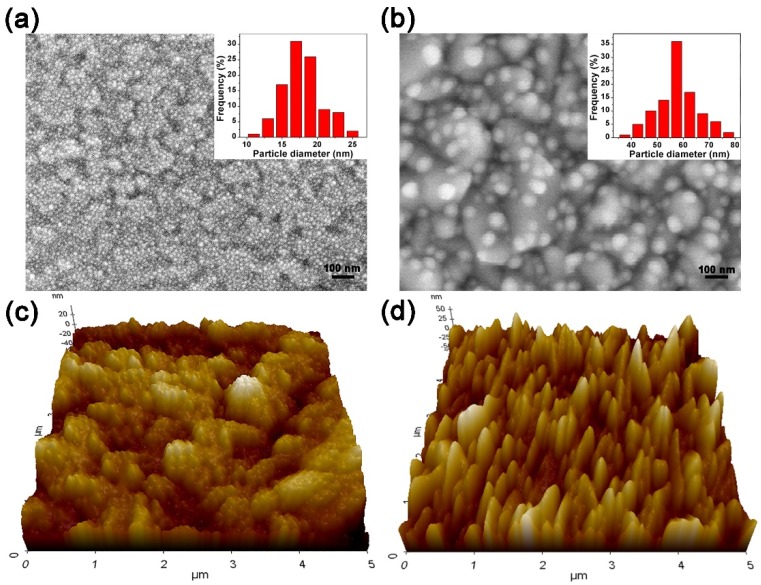
The SEM images of AuNPs (**a**), and TiO_2_/AuNP (**b**) on FTO substrate; 3D AFM surface plots for AuNPs (**c**), and TiO_2_/AuNP (**d**) on the F-doped SnO_2_ (FTO) substrate. (**c**) shows that the FTO surface is very rough (RMS roughness ~17 nm), which provides large surface area and is beneficial to the photocatalysis; and the FTO surface is decorated with small AuNPs. In (**d**), the RMS roughness is 29 nm.

Figure 3 shows the SEM images of the prepared AuNPs film and the AuNPs/TiO_2_ film, respectively. The AuNPs are spherical and widely spread over the surface, with the size mostly in the range of 15–20 nm (see Figure 3a). Deposited by using the ALD, the TiO_2_ film is a conformal, pinhole-free layer (Figure S1, see supplementary information). As the TiO_2_ layer (15 nm thick) is uniformly deposited to cover the AuNPs, it forms TiO_2_/AuNP bumps with the size from 45 to 55 nm (Figure 3b), which is significantly larger than the size of AuNPs. This is attributed to the aggregation of AuNPs during the TiO_2_ annealing and the semi-shell coverage of TiO_2_ on the AuNPs. Figure 3c,d shows the 3D AFM surface plots of AuNPs and TiO_2_/AuNP on the FTO substrate. It can be seen from Figure 2c that the FTO surface is indeed very rough (as expected for large SSA) and is decorated with well-spread small AuNPs. In Figure 3d, large particles appear after depositing TiO_2_. The root mean squared (RMS) roughnesses of the AuNPs layer on the FTO substrate and the TiO_2_/AuNP film on the FTO substrate are 17 and 29 nm, respectively. The AFM results are consistent with the SEM images.

**Figure 4:**
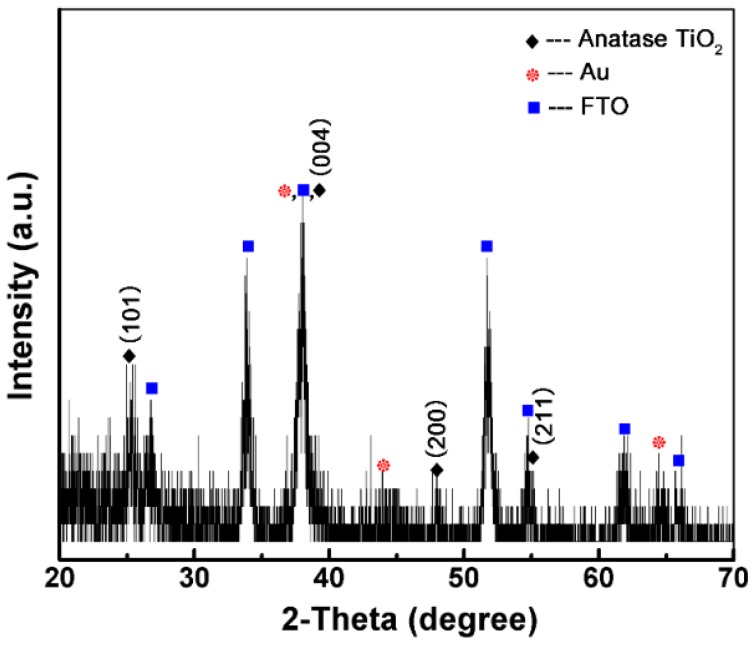
XRD spectra of the annealed TiO_2_/AuNP film.

Figure 4 shows the X-ray diffraction (XRD) patterns of annealed TiO_2_/AuNP film, indicating that the as-prepared nanocomposite is polycrystalline. The diffraction peaks at 2*θ* = 25.3°, 37.8°, 48.05° and 55.1° well match the (101), (004), (200) and (211) planes of the anatase structure of TiO_2_ (JCPDS file no. 21-1272). The peaks at 2*θ* = 37.8°, 44.39°, and 64.58° are the crystal planes of Au (JCPDS file no. 04-0784) [29,30].

**Figure 5:**
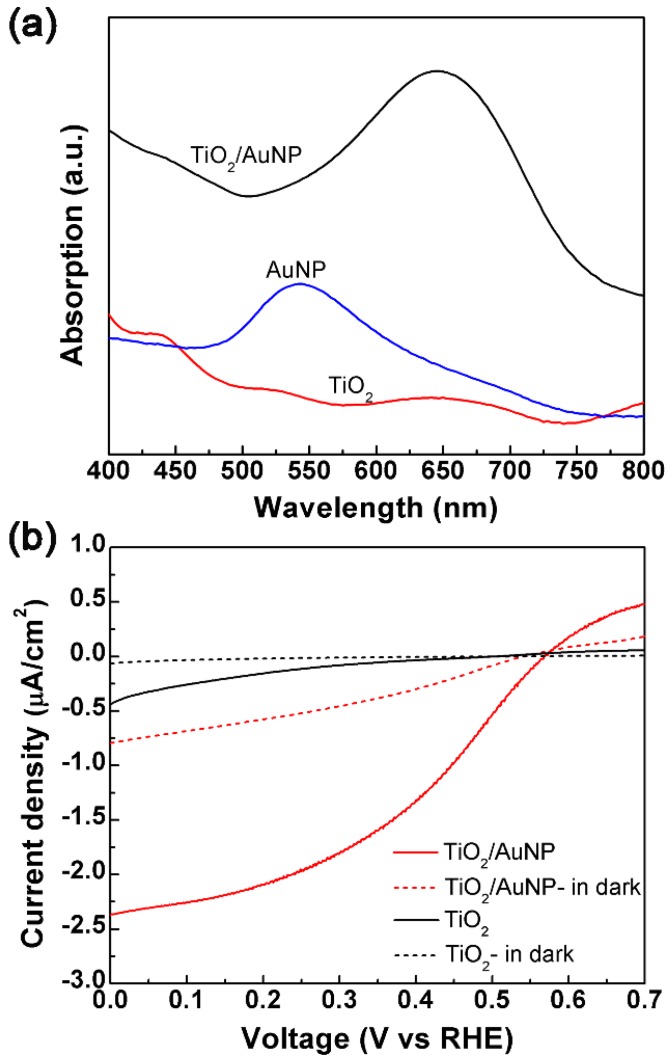
(**a**) Absorption spectra of the bare TiO_2_ film, the AuNP film and the TiO_2_/AuNP film, all are on the FTO substrate. (**b**) The measured *I-V* curves of the TiO_2_ film and the TiO_2_/AuNP film.

Figure 5a plots the absorption spectrum of the TiO_2_/AuNP film as compared to those of the bare TiO_2_ film and the AuNP film. All the films are deposited on the FTO substrate. Here, the absorption intensity *A* is calculated by the equation *A* = 1 − *R* − *T*, where *R* and *T* represent the normalized reflection intensity and the normalized transmission intensity, respectively. The bare TiO_2_ film has very low absorption and shows no obvious peak in visible light. The AuNP film presents an increased absorption with the peak at 550 nm. In contrast, the TiO_2_/AuNP film shows a much stronger and broader absorption over 400–800 nm, with a peak at 650 nm, which is coherent with the previous research [31,32]. The redshift of the TiO_2_/AuNP absorption peak is caused by the larger refractive index of the TiO_2_ (2.52 for anatase TiO_2_ as compared to 1 for air) [27]. The broader absorption peak is due to the modification of electronic states which caused by the heterojunction-induced charge transfer interaction [29]. It also indicates that the TiO_2_ is in anatase crystalline phase, which is consistent with the result in XRD spectra results [32]. In the other words, the TiO_2_/AuNP film has much enhanced absorption over the whole visible light range.

To further support the LSPR effect of AuNPs, photocurrent experiments are carried out in a standard three-electrode system, consisting of the working electrode, the Ag/AgCl reference electrode, and the Pt wire counter electrode. This work uses an electrochemical station (CHI 660E, Shanghai Chenhua Co., Ltd. China). The *I-V* curves of the TiO_2_ film and the TiO_2_/AuNP film in Figure 5b are obtained in the Na_2_SO_4_ electrolyte (0.5 M) under the irradiation of a Xe lamp (300 mW/cm^2^) fixed with a UV filter (cut-off wavelength 420 nm). For control, the photocurrent curves of the films in a dark environment (i.e., with no irradiation) are plotted as well. It is obvious that the photocurrent is largely enhanced for the TiO_2_/AuNP film as compared to the bare TiO_2_ film. With the AuNPs, the TiO_2_/AuNP film under visible light produces a larger change of the photocurrent, about five times of that of the TiO_2_ at an applied bias of 0 V vs. RHE.

### 3.2. Photodegradation Performance

**Figure 6:**
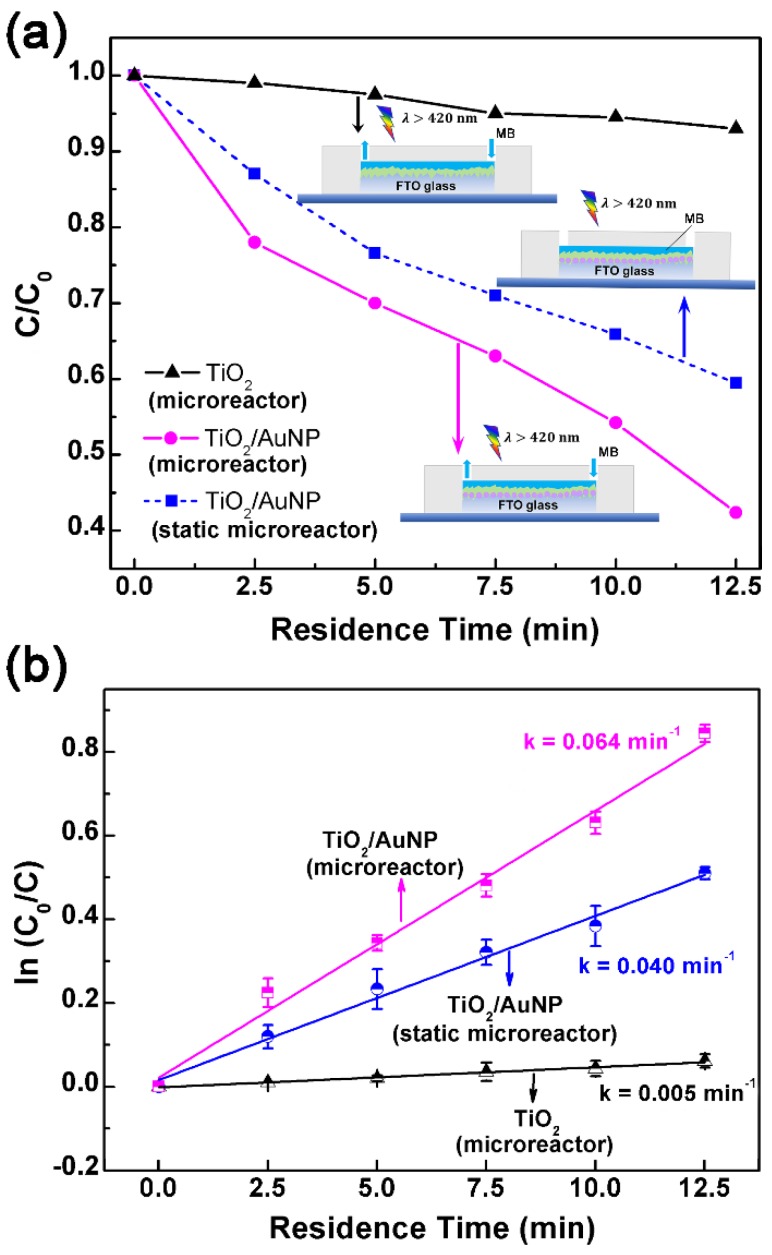
(**a**) Measured MB degradation curve and (**b**) pseudo-first-order kinetics of the photodegradation using three different reactors: the microreactor with the TiO_2_ film, the static microreactor with the TiO_2_/AuNP film and the running microreactor with the TiO_2_/AuNP film. The irradiation is the visible light with > 420 nm.

Figure 6a shows the timeline of photodegradation of MB for three different catalysts: the TiO_2_ film in the microreactor, the TiO_2_/AuNP film in the static microreactor (i.e., the solution in the microchamber is not moving), and the TiO_2_/AuNP film in the microreactor. The degradation is represented by the ratio C/C_0_, where C_0_ and C are the initial MB concentration and the MB concentration at the given time, respectively. In the microreactors, the effective residence time of the MB solution in the reaction chamber is related to the flow rate by the relationship [33]:

Effective residence time = (chamber volume)/(flow rate),(1)

Here, the chamber volume is 50 μL.

In the measurement, the flow rates of the syringe pump are set to be 20, 10, 6.7, 5, and 4 μL/min, corresponding to the residence times of 2.5, 5, 7.5, 10 and 12.5 min, respectively. To quantify the influence of flow movement, a static microreactor with the same TiO_2_/AuNP film is used as the reference. The same amount of the MB solution (50 μL) is added to the static microreactor and then irradiated under the same solar light for 2.5, 5, 7.5, 10 and 12.5 min, separately. The only difference is that the solution in the static microreactor is kept stationary (i.e., not driven to move by the external syringe pump).

It is clearly seen from Figure 6a that the MB degradation increases with a longer residence time. After 12.5 min of light irradiation, 58% of the MB solution is degraded by the TiO_2_/AuNP microreactor while only 7% of the MB solution is degraded by the TiO_2_ microreactor. However, 41% of the MB solution is degraded by the TiO_2_/AuNP static microreactor in 12.5 min. The TiO_2_/AuNP microreactor exhibits faster MB degradation than the static microreactor. This is because the static microreactor lacks a flow movement and causes a slow diffusion of MB molecules, which eventually limits the photocatalytic efficiency. In the other words, the flow motion in the microreactor is actually beneficial to the photodegradation. In comparison to the TiO_2_/AuNP microreactor and the TiO_2_/AuNP static microreactor, the TiO_2_ microreactor shows the lowest degradation efficiency, which verifies the significant contribution of AuNPs to the photocatalytic reaction in visible light region.

Figure 6b plots the pseudo-first-order kinetics of the MB degradation. Each data point is repeated three times. The efficiency of MB photodegradation is determined quantitatively using the pseudo-first-order model [34]:

(2) $$\ln\left(\frac{C_{0}}{C}\right)=kt,$$

where *k* represents the reaction rate constant and *t* is the residence time.

The constant *k* measures 0.005, 0.040, and 0.064 min^−1^ for the TiO_2_ microreactor, the TiO_2_/AuNP static microreactor and the TiO_2_/AuNP microreactor, respectively. Correspondingly, the TiO_2_/AuNP microreactor presents an enhancement factor of 13 with respect to the TiO_2_ microreactor and 1.6 to the TiO_2_/AuNP static microreactor. In the other words, the AuNP contributes 13-fold enhancement and the flow motion yields a 1.6-fold enhancement in the reaction rate constant. As mentioned in the absorption spectra section, the AuNPs exhibit significant optical absorption and scattering properties due to the existence of LSPR. When the AuNPs are covered and screened by the TiO_2_ thin layer_,_ the hot electrons generated from the LSPR excitation of AuNPs are injected into the conduction band of TiO_2_ to produce O_2_^•−^ radicals in visible light [35,36]. The reactive oxygen species O_2_^•−^ is a highly potent oxidizing agent for the degradation of methylene blue molecules.

**Figure 7:**
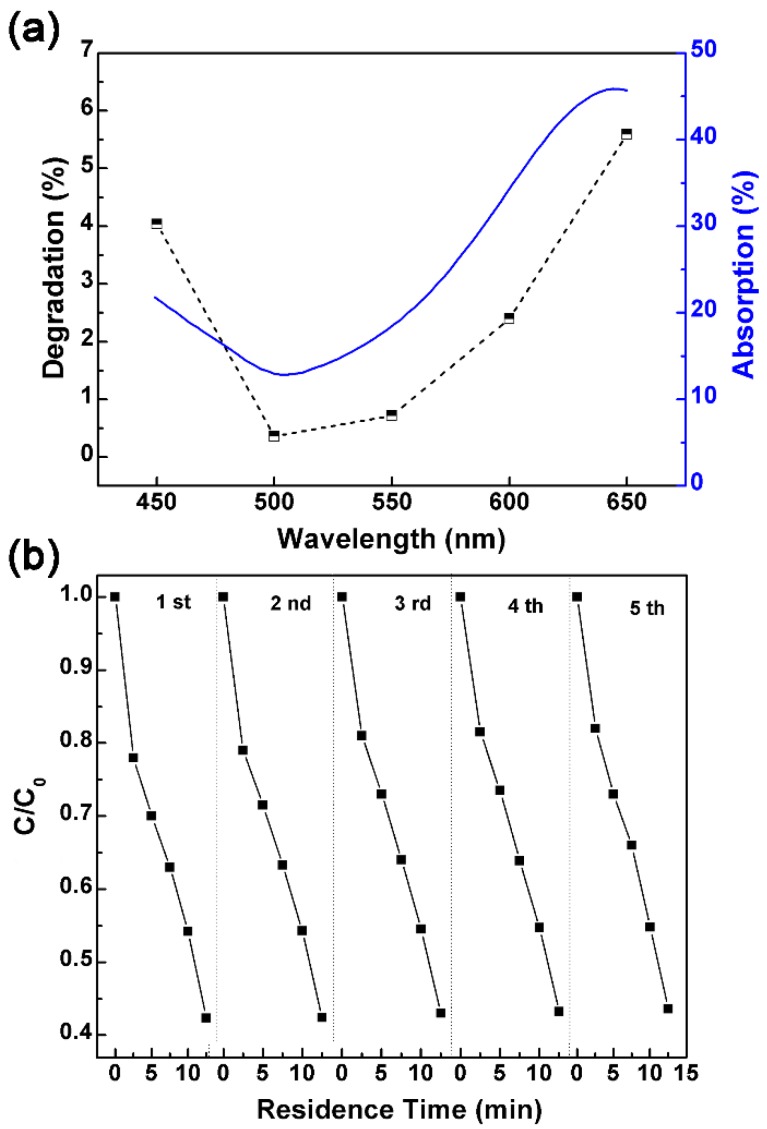
(**a**) MB decomposition efficiency for TiO_2_/AuNP microreactor under five different illumination light in visible light. The y axis title “Degradation” represents (1 − *C*/*C*_0_) × 100%. (**b**) Performance of repeated tests of the TiO_2_/AuNP microreactor under *λ* > 420 nm illumination.

To further confirm the visible-light-driven photocatalytic decomposition of MB is a result of the plasmonic hot carriers, we have conducted a set of control experiments by illuminating the same microfluidic devices with monochromatic light of different wavelengths. Figure 7a plots the MB degradation (1 − *C*/*C*_0_) of the TiO_2_/AuNP microreactor under the visible light of five different wavelengths (i.e., 450, 500, 550, 60, and 650 nm). For easy comparison, the absorption spectrum extracted from Figure 5 is plotted as well. It is seen that the degradation efficiency follows the same trend as the absorption spectrum, proving that the photodegradation results from the absorption, which in turn originates from the plasmonic effect of AuNPs. 

To examine the reusability and stability of the TiO_2_/AuNP microreactor, the photodegradation curves under visible light irradiation are Figure 7b for five repeated tests. After five cycles, the degradation remains as high as 57% in 12.5 min, and no significant drop is observed in the photodegradation activity. This indicates that the TiO_2_/AuNP microreactor is of good stability and can be reused, i.e., it is readily applicable for continuous water treatment.

## 4. Conclusions

In summary, we have embedded the TiO_2_/AuNP film in the microreactor to combine the advantages of both the plasmonic effect and the microfluidics, such as strong visible light absorption, large surface area, short diffusion length, fast reaction rate, and easy control of the reaction conditions. Compared with the bare TiO_2_ film, the AuNPs contribute an enhancement factor of 13 to the reaction rate constant and the microfluidic structure yields 1.6. The repeated tests show that the TiO_2_/AuNP microreactor has high stability and reusability, making it promising for the continuous photocatalytic degradation of organic pollutants.

## Supplementary Materials

The following are available online at https://www.mdpi.com/2072-666X/10/12/869/s1, Figure S1: The SEM image (a) and the 3D AFM surface plot (b) of the TiO2 film (thickness 15 nm) on the FTO substrate. In (a), the TiO_2_ film is conformal and pinhole free.

## References

[1] Shi W., Song Y., Zhang X., Duan D., Wang H., Sun Z. (2018). Nanoporous Pt/TiO_2_ nanocomposites with greatly enhanced photocatalytic performance. J. Chin. Chem. Soc..

[2] Van Gerven T., Mul G., Moulijn J., Stankiewicz A. (2007). A review of intensification of photocatalytic processes. Chem. Eng. Process. Process Intensif..

[3] Parmar J., Jang S., Soler L., Kim D.P., Sánchez S. (2015). Nano-photocatalysts in microfluidics, energy conversion and environmental applications. Lab Chip.

[4] Wang N., Lei L., Zhang X.M., Tsang Y.H., Chen Y., Chan H.L.W. (2011). A comparative study of preparation methods of nanoporous TiO_2_ films for microfluidic photocatalysis. Microelectron. Eng..

[5] Ling C.M., Mohamed A.R., Bhatia S. (2004). Performance of photocatalytic reactors using immobilized TiO_2_ film for the degradation of phenol and methylene blue dye present in water stream. Chemosphere.

[6] Zheng Y., Chen C., Zhan Y., Lin X., Zheng Q., Wei K., Zhu J. (2008). Photocatalytic activity of Ag/ZnO heterostructure nanocatalyst: Correlation between structure and property. J. Phys. Chem. C.

[7] Fragua D.M., Abargues R., Rodriguez-Canto P.J., Sanchez-Royo J.F., Agouram S., Martinez-Pastor J.P. (2015). Au-ZnO Nanocomposite Films for Plasmonic Photocatalysis. Adv. Mater. Interfaces.

[8] Zhang X., Chen Y.L., Liu R.-S., Tsai D.P. (2013). Plasmonic photocatalysis. Reports Prog. Phys..

[9] Ho K.H.W., Shang A., Shi F., Lo T.W., Yeung P.H., Yu Y.S., Zhang X., Wong K.Y., Lei D.Y. (2018). Plasmonic Au/TiO_2_-dumbbell-on-film nanocavities for high-efficiency hot-carrier generation and extraction. Adv. Funct. Mater..

[10] Dinh C.T., Yen H., Kleitz F., Do T.O. (2014). Three-dimensional ordered assembly of thin-shell Au/TiO_2_ hollow nanospheres for enhanced visible-light-driven photocatalysis. Angew. Chemie Int. Ed..

[11] Tan F., Wang N., Lei D.Y., Yu W., Zhang X. (2017). Plasmonic black absorbers for enhanced photocurrent of visible-light photocatalysis. Adv. Opt. Mater..

[12] Dijkstra M.F.J., Panneman H.J., Winkelman J.G.M., Beenackers A.A.C.M., Kelly J.J. (2002). Modeling the photocatalytic degradation of formic acid in a reactor with immobilized catalyst. Chem. Eng. Sci..

[13] Mills A., Wang J. (1998). Photomineralisation of 4-chlorophenol sensitised by TiO_2_ thin films. J. Photochem. Photobiol. A Chem..

[14] Huang X., Hao H., Liu Y., Zhu Y., Zhang X. (2017). Rapid screening of graphitic carbon nitrides for photocatalytic cofactor regeneration using a drop reactor. Micromachines.

[15] Lindstrom H., Wootton R., Iles A. (2007). High surface area titania photocatalytic microfluidic reactors. AIChE J..

[16] Wang N., Zhang X., Wang Y., Yu W., Chan H.L.W. (2014). Microfluidic reactors for photocatalytic water purification. Lab Chip.

[17] Wang N., Tan F., Wan L., Wu M., Zhang X. (2014). Microfluidic reactors for visible-light photocatalytic water purification assisted with thermolysis. Biomicrofluidics.

[18] Wang N., Tan F., Tsoi C.C., Zhang X. (2017). Photoelectrocatalytic microreactor for seawater decontamination with negligible chlorine generation. Microsyst. Technol..

[19] Wang N., Zhang X., Chen B., Song W., Chan N.Y., Chan H.L.W. (2012). Microfluidic photoelectrocatalytic reactors for water purification with an integrated visible-light source. Lab Chip.

[20] Zhu Y., Huang Z., Chen Q., Wu Q., Huang X., So P.-K., Shao L., Yao Z., Jia Y., Li Z. (2019). Continuous artificial synthesis of glucose precursor using enzyme-immobilized microfluidic reactors. Nat. Commun..

[21] Matsushita Y., Ichimura T., Ohba N., Kumada S., Sakeda K., Suzuki T., Tanibata H., Murata T. (2007). Recent progress on photoreactions in microreactors. Pure Appl. Chem..

[22] Liu A.L., Li Z.Q., Wu Z.Q., Xia X.H. (2018). Study on the photocatalytic reaction kinetics in a TiO_2_ nanoparticles coated microreactor integrated microfluidics device. Talanta.

[23] Liao W., Wang N., Wang T., Xu J., Han X., Liu Z., Zhang X., Yu W. (2016). Biomimetic microchannels of planar reactors for optimized photocatalytic efficiency of water purification. Biomicrofluidics.

[24] Wootton R.C.R., Fortt R., De Mello A.J. (2002). On-chip generation and reaction of unstable intermediates—Monolithic nanoreactors for diazonium chemistry: Azo dyes. Lab Chip.

[25] Zhu Y., Chen Q., Shao L., Jia Y., Zhang X. (2019). Microfluidic immobilized enzyme reactors for continuous biocatalysis. React. Chem. Eng..

[26] Huang X., Wang J., Li T., Wang J., Xu M., Yu W., El Abed A., Zhang X. (2018). Review on optofluidic microreactors for artificial photosynthesis. Beilstein J. Nanotechnol..

[27] Sakai N., Fujiwara Y., Takahashi Y., Tatsuma T. (2009). Plasmon-Resonance-based generation of cathodic photocurrent at electrodeposited gold nanoparticles coated with TiO_2_ films. ChemPhysChem.

[28] Chen H., Liu G., Wang L. (2015). Switched photocurrent direction in Au/TiO_2_ bilayer thin films. Sci. Rep..

[29] Huang J., He Y., Wang L., Huang Y., Jiang B. (2017). Bifunctional Au@TiO_2_ core–shell nanoparticle films for clean water generation by photocatalysis and solar evaporation. Energy Convers. Manag..

[30] Naik G.K., Mishra P.M., Parida K. (2013). Green synthesis of Au/TiO_2_ for effective dye degradation in aqueous system. Chem. Eng. J..

[31] Della Gaspera E., Karg M., Baldauf J., Jasieniak J., Maggioni G., Martucci A. (2011). Au nanoparticle monolayers covered with sol-gel oxide thin films: Optical and morphological study. Langmuir.

[32] Lee M., Chae L., Lee K.C. (1999). Microstructure and surface plasmon absorption of sol-gel-prepared Au nanoclusters in TiO_2_ thin films. Nanostructured Mater..

[33] Lei L., Wang N., Zhang X.M., Tai Q., Tsai D.P., Chan H.L.W. (2010). Optofluidic planar reactors for photocatalytic water treatment using solar energy. Biomicrofluid..

[34] Lamberti A. (2015). Microfluidic photocatalytic device exploiting PDMS/TiO_2_ nanocomposite. Appl. Surf. Sci..

[35] Su Y.H., Ke Y.F., Cai S.L., Yao Q.Y. (2012). Surface plasmon resonance of layer-by-layer gold nanoparticles induced photoelectric current in environmentally-friendly plasmon-sensitized solar cell. Light Sci. Appl..

[36] Xu J., Gu P., Birch D.J.S., Chen Y. (2018). Plasmon-Promoted Electrochemical Oxygen Evolution Catalysis from Gold Decorated MnO_2_ Nanosheets under Green Light. Adv. Funct. Mater..

