# Supplementary material for: Microfluidic Reactors for Plasmonic Photocatalysis Using Gold Nanoparticles
Source: Micromachines (Basel). 2019 Dec 11;10(12):869. doi: 10.3390/mi10120869 (PMC6952777; doi:10.3390/mi10120869)
Supplement: Supplementary file 1 [file micromachines-10-00869-s001.pdf]

micromachines

# Supplementary Materials: Microfluidic Reactors for Plasmonic Photocatalysis Using Gold Nanoparticles

**Huaping Jia, Yat Lam Wong, Aoqun Jian, Chi Chung Tsoi, Meiling Wang, Wanghao Li, Wendong Zhang, Shengbo Sang and Xuming Zhang**

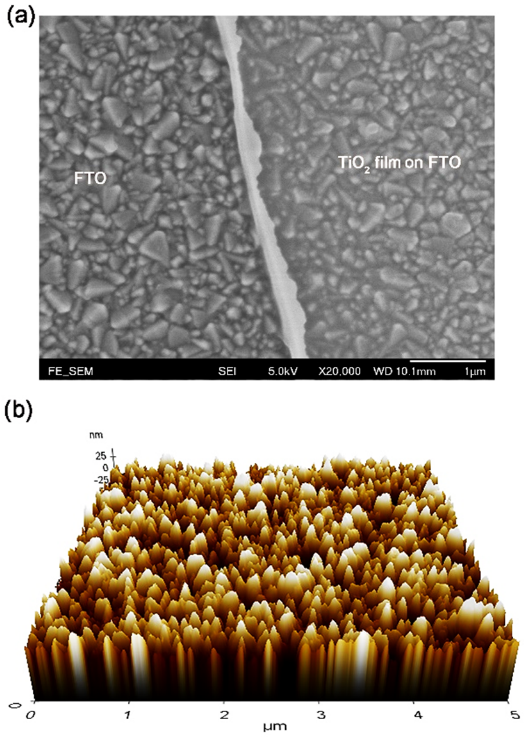


**Figure S1.** The SEM image (**a**) and the 3D AFM surface plot (**b**) of the $TiO_2$ film (thickness 15 nm) on the FTO substrate. In (a), the $TiO_2$ film is conformal and pinhole free.
